# Non-targeted metabolomics reveals metabolic signatures associated with *Clostridioides difficile* virulence

**DOI:** 10.3389/fmicb.2026.1731048

**Published:** 2026-02-26

**Authors:** Huixin Pan, Miao Zhang, Dongxiao Zhao, Qinglu Wang, Hua Shang, Ying Luo

**Affiliations:** 1 Department of Gastroenterology, Zibo Central Hospital, Zibo, China; 2 College of Graduate Education, Shandong Sport University, Jinan, China; 3 Zibo Center for Disease Control and Prevention, Zibo, China; 4 Ningxia Integrated Chinese and Western Medicine Hospital, Yinchuan, China; 5 Department of Clinical Laboratory, Zibo Central Hospital, Zibo, China

**Keywords:** bile acid metabolism, *Clostridioides difficile*, metabolic reprograming, non-targeted metabolomics, virulence gradient

## Abstract

**Background:**

*Clostridioides difficile* (*C. difficile*) is a major pathogen causing antibiotic-associated diarrhea and pseudomembranous colitis, with *Clostridioides difficile* infection (CDI) showing a global upward trend. Significant differences exist in clinical manifestations and pathogenic potential among strains of varying virulence, yet their underlying metabolic basis and molecular mechanisms remain poorly understood. Systematic investigation of metabolic characteristics across strains with differing virulence levels is crucial for elucidating pathogenic mechanisms and identifying potential metabolic targets.

**Methods:**

Four *C. difficile* strains with varying virulence gradients (*RT027/ST1, RT046/ST35, RT017/ST37, RT012/ST54*) were selected. Liquid chromatography-mass spectrometry (LC–MS)-based non-targeted metabolomics was employed, combined with principal component analysis (PCA), Partial Least Squares Discriminant Analysis (PLS-DA), and pathway enrichment analysis to compare metabolic differences among strains.

**Results:**

A total of 3,255 metabolites were identified (1,735 in positive ion mode and 1,520 in negative ion mode). Multivariate statistical models revealed significant metabolic profile separation among the four strains. The highly virulent strain (*ST1*) exhibited significantly enhanced activation in lipid metabolism, bile acid metabolism, nicotinic acid/nicotinamide energy metabolism, and branched-chain amino acid fermentation pathways compared to the low-virulence strain (*ST54*). Analysis of virulence gradient-related metabolites identified 13 differentially expressed metabolites with potential biological significance, including upregulated isomangiferin, ginsenoside ro, glycocholic acid, lactic acid, isovalerate, and downregulated inosine, n-acetylmuramate, n-acetylglucosamine, cholesterol. These metabolites were primarily enriched in pathways involving bile acid synthesis, pyruvate metabolism, amino sugar and nucleotide sugar metabolism, and sterol biosynthesis.

**Conclusion:**

This study systematically characterized the metabolomic profiles of *C. difficile* strains of different ST types, revealing that their enhanced virulence is closely associated with the reprograming of energy metabolism, membrane lipid structural remodeling, and bile acid metabolism. Metabolic differences suggest that highly virulent strains may enhance fermentation and lipid synthesis pathways to gain stronger survival and infection capabilities. The 13 candidate metabolites identified hold promise as potential biomarkers for distinguishing strain virulence levels, providing new theoretical basis for subsequent targeted metabolic regulation and anti-*C. difficile* therapies.

## 1 Introduction

*C. difficile* is a significant bacterial pathogen causing hospital-acquired diarrhea and pseudomembranous colitis. Its rising infection and recurrence rates in recent years have made it a major global public health concern (Jenior et al., 2021; Buddle and Fagan, 2023). Traditionally, its pathogenic mechanisms have focused on the two major toxins it produces, TcdA and TcdB, which exert their primary virulence effects by damaging intestinal epithelial cells and inducing inflammatory responses (Neumann-Schaal et al., 2019; Pourliotopoulou et al., 2024). However, toxin levels alone cannot fully explain the significant virulence differences among strains (Zhang et al., 2025)—for example, some strains exhibit high toxin expression but do not cause the most severe clinical manifestations, while certain low-toxin strains can still induce severe disease under specific host conditions (Hunt and Ballard, 2013; Lanis et al., 2010; Luo et al., 2022). Thus, beyond toxins, strain-specific biological characteristics, metabolic capabilities, spore formation, adhesion/colonization abilities, and interactions with the host microbiome may also be crucial determinants of virulence variation (Hunt and Ballard, 2013; Lanis et al., 2010; Kong et al., 2020).

Recent studies reveal that *C. difficile*’s ability to acquire nutrients, metabolic adaptability, and substrate utilization spectrum within its intestinal niche directly influence its colonization success, spore germination, toxin expression, and competitive advantage against hosts/microbiota (Marshall et al., 2023; Girinathan et al., 2021). Specifically, the bacterium can ferment branched-chain amino acids and utilize alternative pathways for central carbon metabolism, such as pyruvate and acetyl-CoA, thereby gaining metabolic advantages in the intestinal ecosystem following antibiotic-induced disruption of the microbiota (Neumann-Schaal et al., 2015). Simultaneously, metabolic substrates derived from the host or symbiotic bacteria—such as bile acids, sugar alcohols, and mucin degradation products—have been demonstrated as crucial nutritional sources for *C. difficile* colonization and proliferation, indicating that its metabolic adaptation is closely intertwined with host-microbiota interactions (Castro et al., 2025). Recent studies indicate significant metabolic differences between virulent and non-virulent strains: highly virulent strains may activate amino acid fermentation pathways, organic acid production pathways, and even lipid metabolism remodeling, while non-virulent strains may exhibit limitations in metabolic activity or substrate utilization (Marvaud et al., 2019; Hamo et al., 2021). Concurrently, non-targeted metabolomics—a comprehensive technique for detecting intracellular and extracellular metabolite changes—has been successfully applied to various pathogenic bacteria, aiding in elucidating the linkages between strain metabolic states, metabolic pathway reprograming, and pathogenic phenotypes (Jenior and Papin, 2022). However, studies on the metabolomic changes of different *C. difficile* virulence strains under pure culture conditions remain scarce, particularly from a non-targeted metabolomics perspective. The differences in substrate utilization profiles, metabolite accumulation, and their correlation with virulence expression among strains have yet to be systematically elucidated.

Therefore, this study aims to compare the metabolic products of *C. difficile* clinical isolates with different ribotype/sequence types using a non-targeted metabolomics approach. Four ribotype/sequence type lineages were selected because they represent a clinically and epidemiologically established gradient of virulence, ranging from the hypervirulent *RT027/ST1* lineage to the relatively low-virulence *RT012/ST54* lineage, with *RT046/ST35* and *RT017/ST37* occupying intermediate positions (Orozco-Aguilar et al., 2020; Luo et al., 2018).

## 2 Materials and methods

### 2.1 Samples and collection

The strains of *C. difficile* strains that were used in the study were picked among a previously acquired collection of clinical isolate that we kept in our laboratory. This group was initially produced via a multicenter epidemiological study which was done at Zibo Central Hospital (ZCH) and the Affiliated Hospital of Qingdao University (AHQU) during March 2016 to April 2017 in which 504 non-repetitive stool samples were taken up in patients with a clinically confirmed CDI. Based on this collection, isolates that represent four big ribotype/sequence type lineages *(RT027/ST1, RT046/ST35, RT017/ST37*, and *RT012/ST54)* were pulled down and utilized in the current experiments on metabolomics. This study did not have any new clinical sampling. The clinical information corresponding to each isolate, including disease severity classification, is summarized in Supplementary Table 1. Between March 2016 and April 2017, a total of 504 non-repetitive stool samples were collected from patients presenting with symptoms of CDI (Luo et al., 2018).

### 2.2 Cultivation and identification of *C. difficile*

Fecal samples were inoculated onto ChromID *C. difficile* agar (CDIF, bioMérieux) and incubated anaerobically at 37°C for 48 h. Typical *C. difficile* colonies were subsequently identified using Matrix-Assisted Laser Desorption/Ionization Time-of-Flight Mass Spectrometry (MALDI-TOF MS) and the VITEK MS system (bioMérieux).

### 2.3 Multilocus sequence typing and PCR ribotyping

MLST was performed by using seven gene loci (adk, atpA, dxr, glyA, recA, soda, and tpi), as previously described Griffiths et al. (Griffiths et al., 2010). PCR products were purified and sequenced at Taihe Biotechnology Company (Beijing, China). DNA sequences were queried against the PubMLST database^1^ to obtain the allele numbers, sequence types (STs) and clades.

PCR ribotyping was performed by capillary gel electrophoresis as previously described (Indra et al., 2008). Gene Marker V2.2.0 (Soft Genetics, America) was used to determine the size of each peak, and ribotypes (RTs) were assigned by presenting the data on the WEBRIBO database^2^ and compared with results reported by Cheng et al. (2016).

### 2.4 Extraction and processing of bacterial metabolome samples

The metabolomic analysis of bacterial cells cultured on *C. difficile* strains *(RT027/ST1*, *RT046/ST35*, *RT017/ST37*, and *RT012/ST54)* was conducted through liquid chromatography mass spectrometry (LC–MS). Upon 48 h of anaerobic growth, bacterial pellets were centrifuged to extract the metabolites.

The bacterial pellets were briefly thawed at 4°C and extracted by adding 200 μL of pre-chilled ultrapure water and 800 μL of pre-chilled methanol. The samples were vortexed thoroughly and subjected to ultrasonic disruption in an ice bath for 20 min. Protein precipitation was achieved by incubation at −20°C for 1 h, followed by centrifugation at 16,000 g and 4°C for 20 min. The resulting supernatants were collected for metabolite analysis.

The rest of the protein pellets were dried at air and resuspended in 200 μL of SDT lysis buffer and the bicinchoninic acid (BCA) assay was used to determine the total biomass of the bacterium. The same volumes of the metabolite-containing supernatants based on the protein content were liquefied and evaporated dry in a vacuum concentrator. The dried extracts before LC–MS analysis were resuspensed with 40μL of the methanol-water solution (1:1, v/v) and centrifuged at the rate of 20,000 g at 4°C for 20 min. The remaining supernatants were pooled to be injected and analyzed later into the LC-MS.

### 2.5 Sample grouping and statistical analysis

In the case of a metabolomic analysis, each sequence type (*ST1*, *ST35*, *ST37*, and *ST54*) were analyzed as independent clinical isolates, with biological repeats. LC–MS analysis was done independently on each of the isolates. To perform a multivariate analysis, matrices of normalized peak intensities were analyzed with principal component analysis (PCA) and partial least squares-discriminant analysis (PLS-DA) with MetaboAnalyst 6.0. Metabolites whose difference in expression between the predefined virulence gradient (*ST54-ST35/ST37-ST1*) could be detected using Spearman correlation analysis were filtered by fold-change.

Metabolites having the ρ > 0.6 and the *P*> 0.05 were regarded as significant with the increase or decrease in virulence. False discovery rate (FDR) invalid on multiple testing correction with the Benjamini–Hochberg correction was applied where necessary. To perform pairwise comparisons depicted in Figures 7, 8, the statistical significance was evaluated with the help of the one-way ANOVA and the *post-hoc* test. Data are presented as mean ± SD.

### 2.6 Liquid chromatography-mass spectrometry analysis conditions

The metabolomics detection platform utilizes a Shimadzu Nexera X2 ultra-high-performance liquid chromatography (UHPLC) system coupled with an AB Sciex Triple TOF 6600 high-resolution mass spectrometers. The chromatographic column employed is a Waters ACQUITY UPLC^®^ HSS T3 column (2.1 × 100 mm, 1.8 μm). Mass spectrometry analysis is performed in positive ion mode with a scan range set at m/z 50–1,000. MS/MS spectra are acquired using data-dependent acquisition (DDA) mode. Specific chromatographic mobile phases and gradient elution programs can be found in Supplementary material.

### 2.7 Data processing and metabolite identification

Raw mass spectrometry data underwent peak extraction, retention time correction, and peak area normalization using MS-DIAL software. Metabolite identification was performed based on MS/MS fragment information, with reference to public databases such as HMDB, KEGG, and MassBank for comparison and screening. To ensure data quality, blank controls and pooled QC samples were incorporated throughout the process for quality control and instrument stability monitoring.

### 2.8 Statistical analysis

Differential metabolites between strains were identified using one-way ANOVA followed by *post hoc* multiple comparison testing. *P*-values were corrected for multiple testing using the Benjamini–Hochberg false discovery rate (FDR). Metabolites with FDR-adjusted *P* < 0.05 were considered statistically significant.

## 3 Results

### 3.1 Establishment and evaluation of metabolome profiles

**FIGURE 1:**
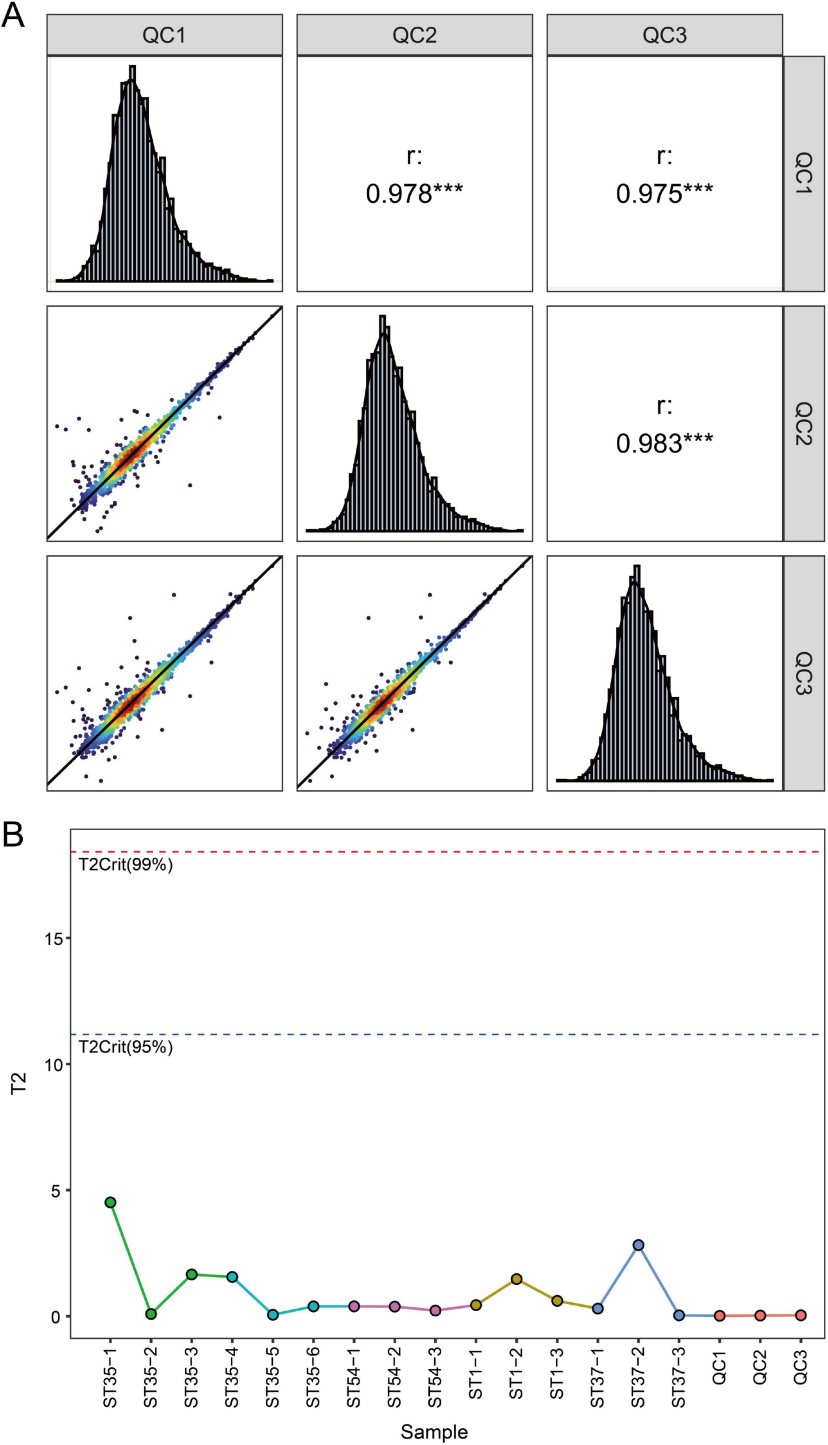
Heatmap of quality control (QC) sample correlations. **(A)** Pairwise Pearson correlation analysis among QC samples (QC1, QC2, QC3). **(B)** Hotelling’s T^2^ plot of all samples under the multivariate model. ***P < 0.001.

**FIGURE 2:**
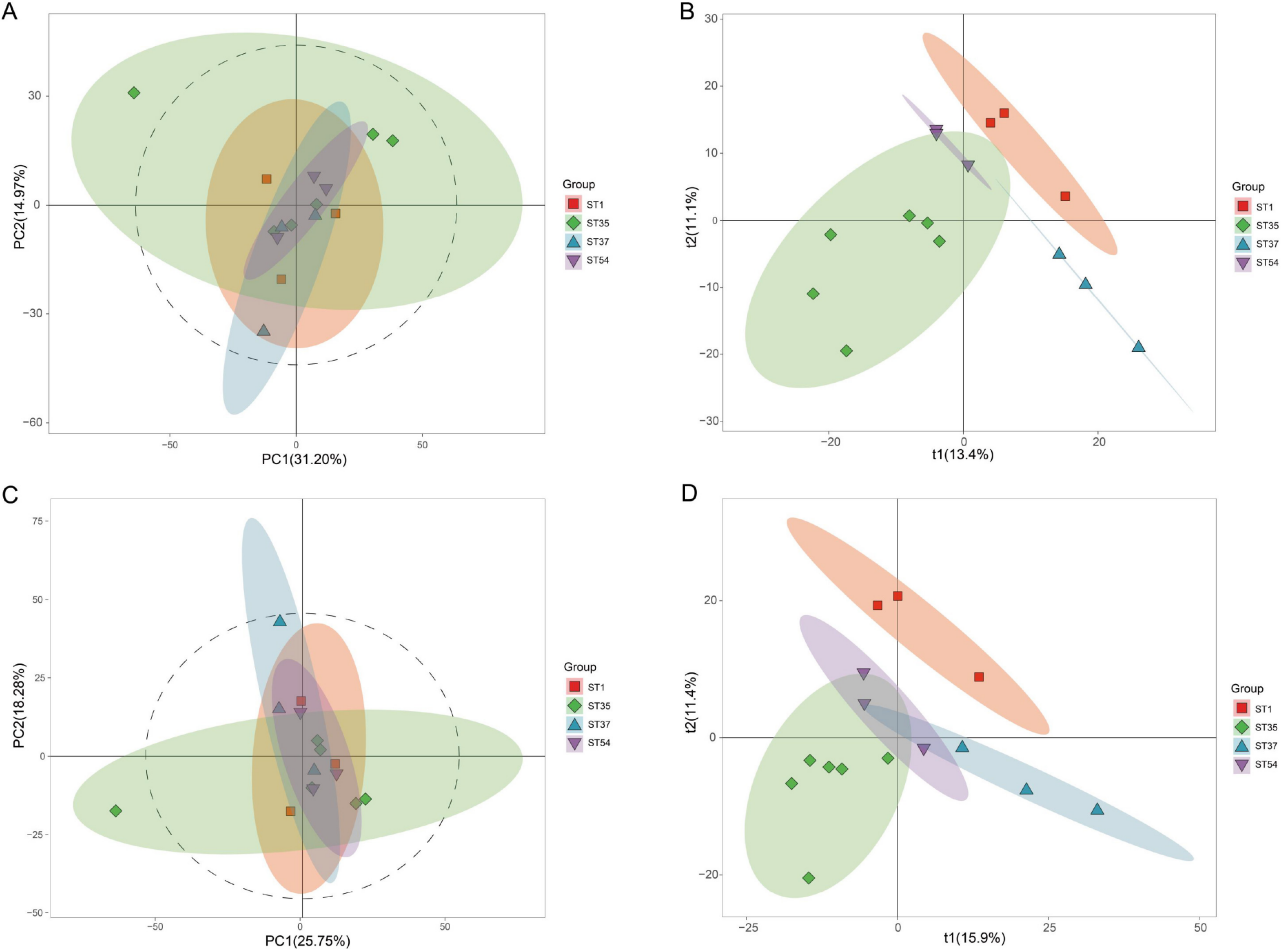
Multivariate statistical analysis results for different *C. difficile* strains. **(A)** Principal component analysis (PCA) score plot in positive ion mode. **(B)** Partial least squares discriminant analysis (PLS-DA) score plot in positive ion mode. **(C)** PCA score plot in negative ion mode. **(D)** PLS-DA score plot in negative ion mode. Results reveal a distinct separation trend among the four strains with varying virulence gradients *(ST1, ST35, ST37, ST54)*, suggesting unique metabolic profiles for each strain.

This study employed LC-MS non-targeted metabolomics technology to analyze metabolites from four *C. difficile* strains (*RT027/ST1, RT046/ST35, RT017/ST37, RT012/ST54*). Quality control validation demonstrated high consistency in QC samples under combined positive/negative ion modes (mean correlation coefficient > 0.97), indicating stable instrument performance (Figure 1). To further evaluate overall metabolic differences among strains and assess model reliability, principal component analysis (PCA) and partial least squares discriminant analysis (PLS-DA) were performed on data from positive and negative ion modes, respectively. Multivariate statistical analysis revealed significant differences in the metabolite profiles among the four *C. difficile* strains. In the positive ion mode, the PCA score plot (Figure 2A) showed a clear separation trend among the four groups, with principal component 1 (PC1) and principal component 2 (PC2) contributing 31.20 and 14.97% of the variance, respectively. This natural clustering trend was further reinforced and validated in the supervised PLS-DA model (Figure 2B), which demonstrated excellent intergroup separation, indicating unique metabolic phenotypic characteristics among the strains. Similarly, in negative ion mode, the PCA model (Figure 2C, PC1 = 25.75%, PC2 = 18.28%) and the PLS-DA model (Figures 2C,D) both revealed significant intergroup separation. This trend corroborates the positive ion mode analysis, confirming that *C. difficile* strains of different ribotypes (RT) and sequence types (ST) exhibit fundamental differences in their intrinsic metabolite compositions. In summary, the quality control system demonstrated excellent stability, and multivariate statistical models clearly revealed overall differences in the metabolic profiles of *ST1*, *ST35*, *ST37*, and *ST54* strains. This indicates that the metabolomics data obtained in this study are reliable and suitable for subsequent differential metabolite screening and pathway analysis.

### 3.2 Metabolite classification and pathway distribution

**FIGURE 3:**
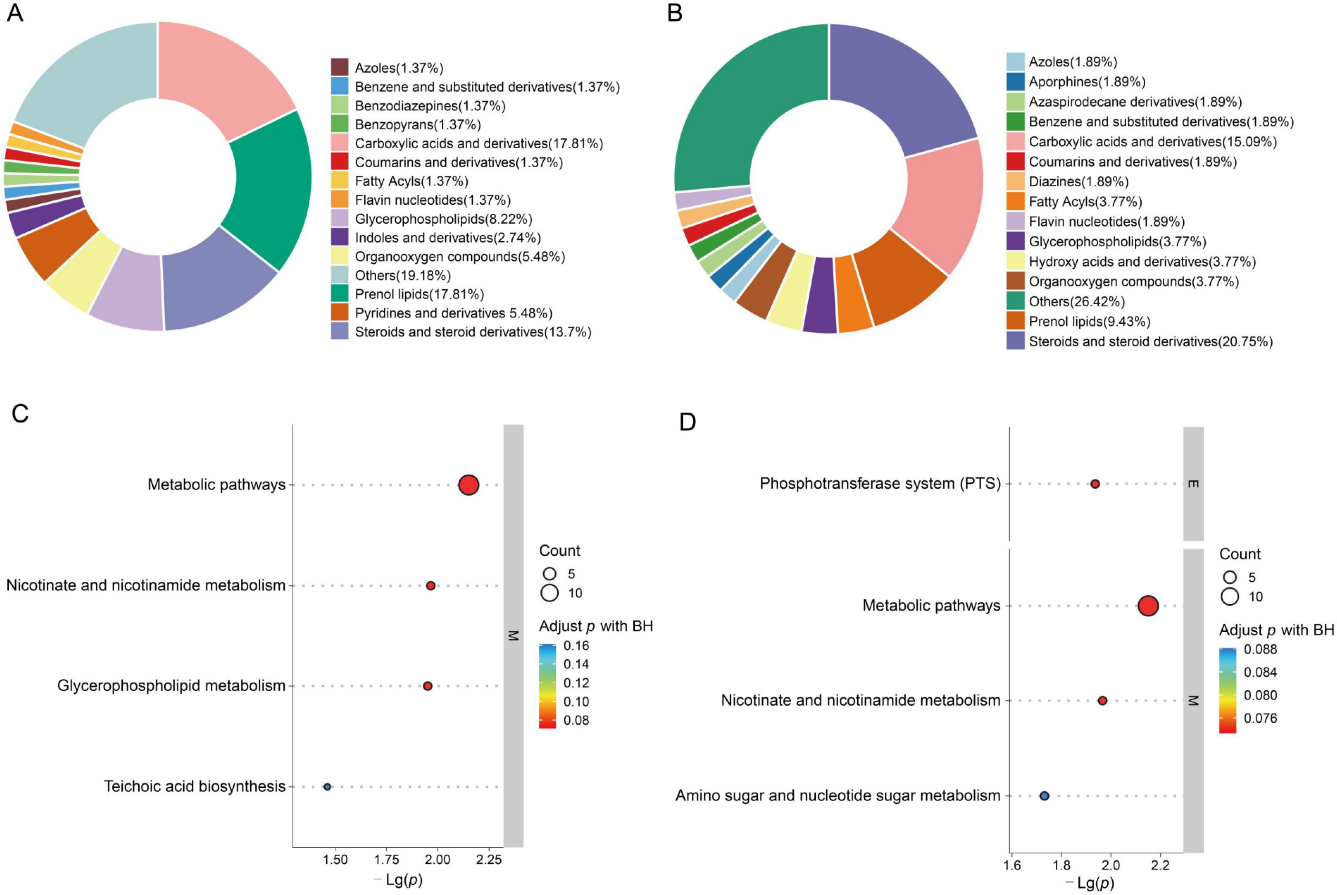
Metabolite classification and KEGG pathway enrichment analysis. The TOP30 KEGG pathways were selected based on their statistical significance and pathway impact scores, representing the most biologically relevant enriched metabolic pathways. **(A)** Chemical classification distribution of metabolites in cationic mode. **(B)** Chemical classification distribution of metabolites in anionic mode. **(C)** Top 30 significantly enriched metabolic pathways in cationic mode (TOP30). **(D)** Top 30 enriched pathways in the anion mode. Results indicate that the cation mode primarily involves lipid metabolism and membrane structural remodeling, while the anion mode predominantly focuses on carbohydrate and sugar metabolism.

This study identified 3,255 metabolites in non-targeted metabolomics analysis, including 1,735 detected in positive ion mode (POS) and 1,520 in negative ion mode (NEG). In the positive ion mode, carboxylic acids and their derivatives, along with isoprenolides, accounted for the highest proportion, followed by steroid-like derivatives and their analogues (Figure 3A). In the negative ion mode, steroids and their derivatives constituted the largest group, followed by carboxylic acids and their derivatives (Figure 3B). KEGG metabolic pathway enrichment analysis (TOP30) revealed that cationic mode results highlighted lipid metabolism and cell membrane remodeling (Figure 3C), while anionic mode emphasized carbohydrate uptake and cell wall carbohydrate metabolism (Figure 3D). These pathways were further interpreted in the Discussion in the context of virulence-associated metabolic reprograming.

### 3.3 Differential metabolite screening and identification

**FIGURE 4:**
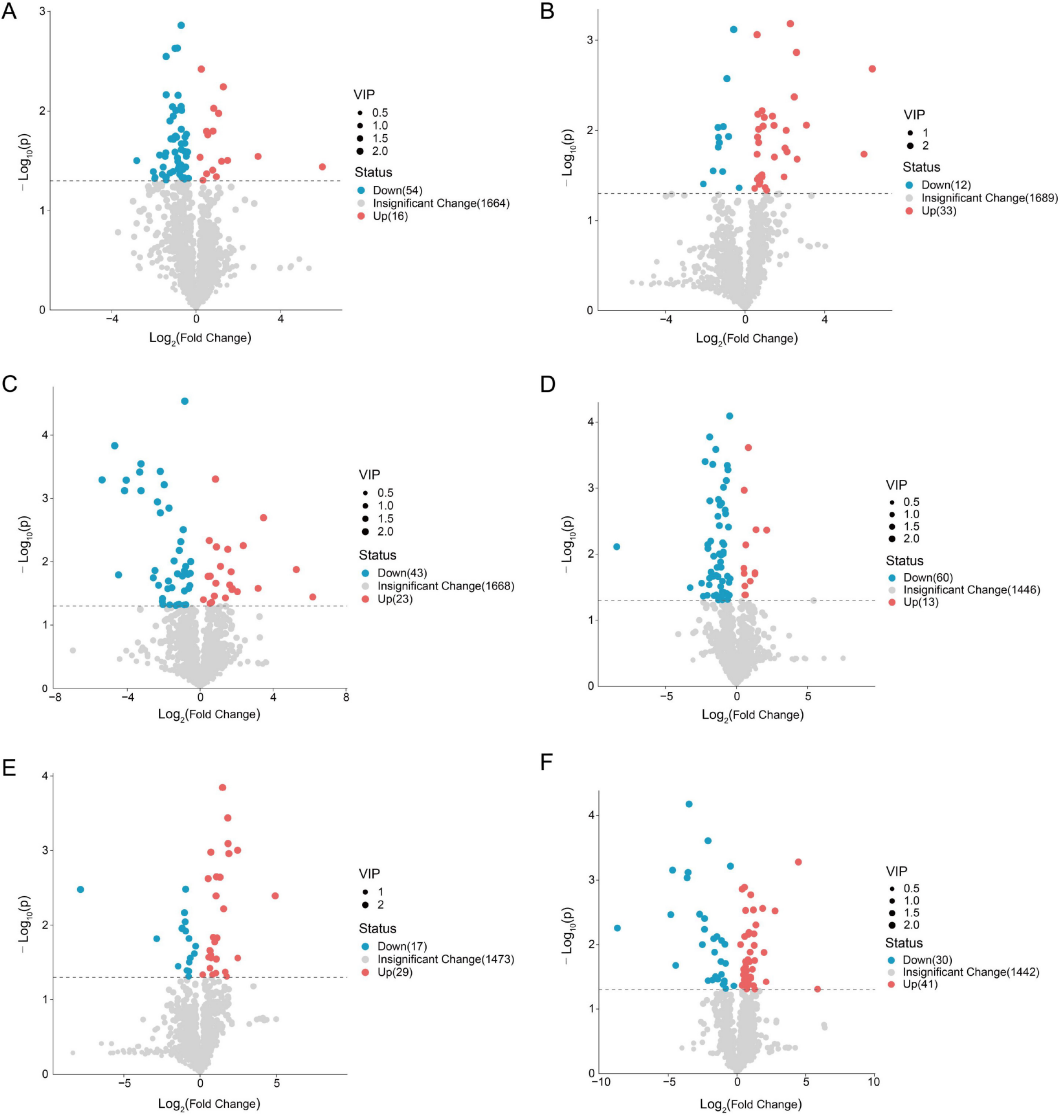
Volcano plot of differentially expressed metabolites among bacterial strains. **(A–C)** Distribution of differentially expressed metabolites in cationic mode across groups *(ST1* vs. *ST54*, *ST1* vs. *ST35*, *ST1* vs. *ST37)*. **(D–F)** Distribution of differentially expressed metabolites in anionic mode across groups. Red dots indicate upregulated metabolites; blue dots indicate downregulated metabolites. Results show that the highly virulent strain (*ST1*) exhibits significant upregulation in pathways related to lipids, bile acids, and energy metabolism.

**FIGURE 5:**
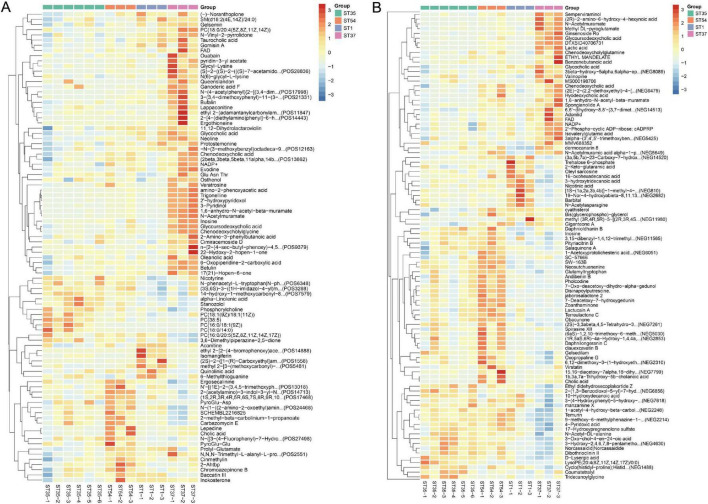
Clustering heatmap of differentially expressed metabolites among strains with different virulence. **(A)** Heatmap generated from metabolites detected in positive ion mode. **(B)** Heatmap generated from metabolites detected in negative ion mode. Rows represent metabolites and columns represent individual strains (*ST35, ST54, ST1, ST37*). Color intensity reflects relative abundance after normalization (red, higher abundance; blue, lower abundance).

Volcano plots (Figure 4) and heatmaps (Figure 5) visually display differential metabolites across different bacterial strains in both positive and negative ion modes. Differential metabolite screening results indicate that 70 significantly differential metabolites were identified in the comparison between ST1 and ST54 in the positive ion mode, including 16 upregulated and 54 downregulated metabolites (VIP > 1.0, *p* < 0.05, FC ≥ 1.5 or FC ≤ 1/1.5) (Figure 4A). Comparing *ST1* and *ST35* revealed 45 differential metabolites: 33 up-regulated and 12 down-regulated. Comparing *ST1* and *ST37* identified 66 differential metabolites: 23 up-regulated and 43 down-regulated (Figures 4B,C). In the anion mode, significant intergroup differences were also observed, with the most abundant differences detected between *ST1* and *ST54*, revealing 73 differentially metabolized compounds (13 up-regulated and 60 down-regulated) (Figures 4D–F).

**FIGURE 6:**
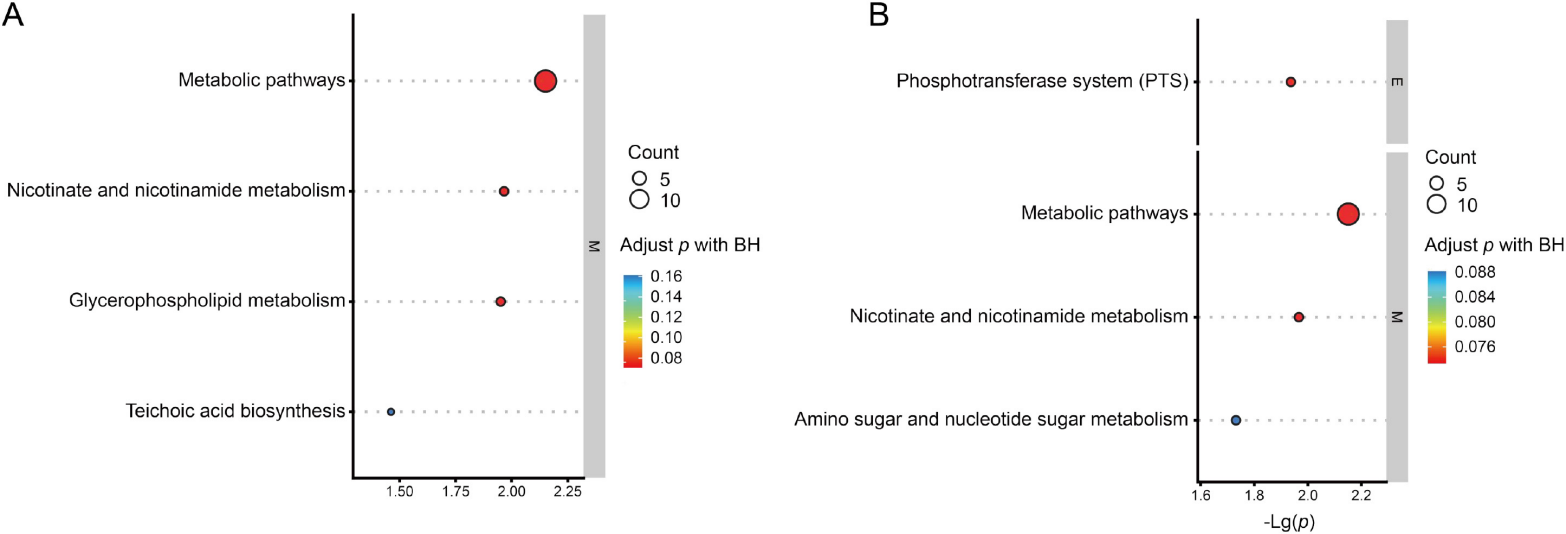
KEGG metabolic pathway enrichment analysis of differentially expressed metabolites. **(A)** Enriched pathways identified in positive ion mode. **(B)** Enriched pathways identified in negative ion mode. The x-axis represents –log10 (*P*-value). Bubble size corresponds to the number of enriched metabolites (count), and color indicates adjusted *P*-value (Benjamini–Hochberg correction).

Metabolic pathway enrichment analysis of differential metabolites revealed that the cationic pattern predominantly highlighted lipid metabolism and cell membrane structural remodeling, suggesting that membrane structure and signal transduction may be key factors underlying virulence differences. The anionic pattern emphasized carbohydrate uptake and utilization (PTS system) alongside cell wall carbohydrate metabolism, suggesting significant differences in energy metabolism and cell wall structural assembly between serotypes. The shared enrichment of nicotinic acid and nicotinamide metabolic pathways indicates that energy metabolism reprograming may underlie the maintenance of distinct virulence levels (Figure 6).

### 3.4 Screening of differentially expressed metabolites associated with virulence gradient and functional enrichment analysis

Based on published epidemiological data, experimental infection models, and the clinical severity scores of the original patient isolates, the four strains were classified into a predefined virulence hierarchy: *ST1 (RT027)* as a high-virulence lineage, *ST35 (RT046)* and *ST37 (RT017)* as intermediate-virulence lineages, and *ST54 (RT012)* as a low-virulence lineage. All analyses referring to “increasing virulence” in this study follow this specific ranking: *ST*54→*ST*35/*ST*37→*ST*1. Using non-targeted metabolomics data of the four *C. difficile* strains that fell in the established virulence gradient (*ST*54→*ST*35/*ST*37→*ST*1), we were able to determine 25 progressive- upregulated and 48 progressive- downregulated metabolites that occurred as the different strains increased in virulence in the positive and negative ion mode. Notably, the metabolites did not vary homogenously across all strains but had monotonic patterns with regard to their relative levels of virulence. As an example, organic acids, steroids, triterpenoids, as well as energy-related metabolites were gradually progressively raised as low virulence *ST54* progressed to intermediate *ST35* and *ST37* to the extremely virulent *ST1*. This trend indicates the activation of pathways linked to energy metabolism, membrane lipid metabolism, and defense against stress are upregulated as strains increase in virulence, which could provide a higher biosynthetic capacity and environmental flexibility to extremely virulent strains.

Conversely, 48 metabolites, including nucleosides and their derivatives, cell wall precursors, bile acid metabolites, and various alkaloids, exhibited a stepwise decrease from *ST54* to *ST1*. This coordinated downregulation along the virulence gradient likely reflects suppressed cell wall biosynthesis and nucleotide metabolism in highly virulent strains, indicating metabolic redistribution toward pathways that support rapid growth, toxin production, and stress resistance.

**FIGURE 7:**
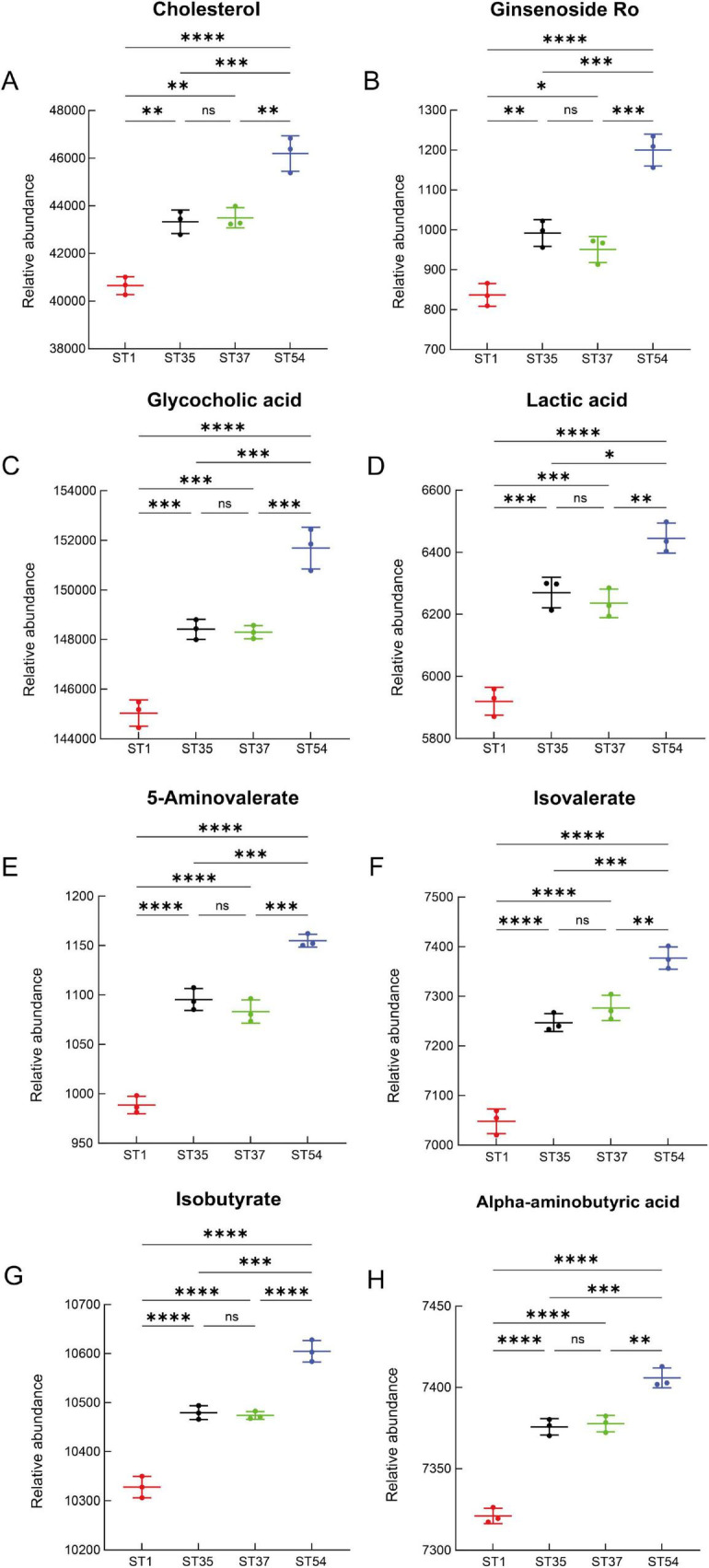
Differentially upregulated metabolites along the virulence gradient. **(A)** Cholesterol. **(B)** Ginsenoside Ro. **(C)** Glycocholic acid. **(D)** Lactic acid. **(E)** 5-Aminovalerate. **(F)** Isovalerate. **(G)** Isobutyrate. **(H)** Alpha - Aminobutyric acid.Relative abundance levels are shown for each strain group. Data are presented as mean ± SD. Statistical significance is indicated as ns (not significant), *P < 0.05, **P < 0.01, ***P < 0.001, ****P < 0.0001. *P*-values were adjusted for multiple testing using the Benjamini-Hochberg false discovery rate (FDR).

**FIGURE 8:**
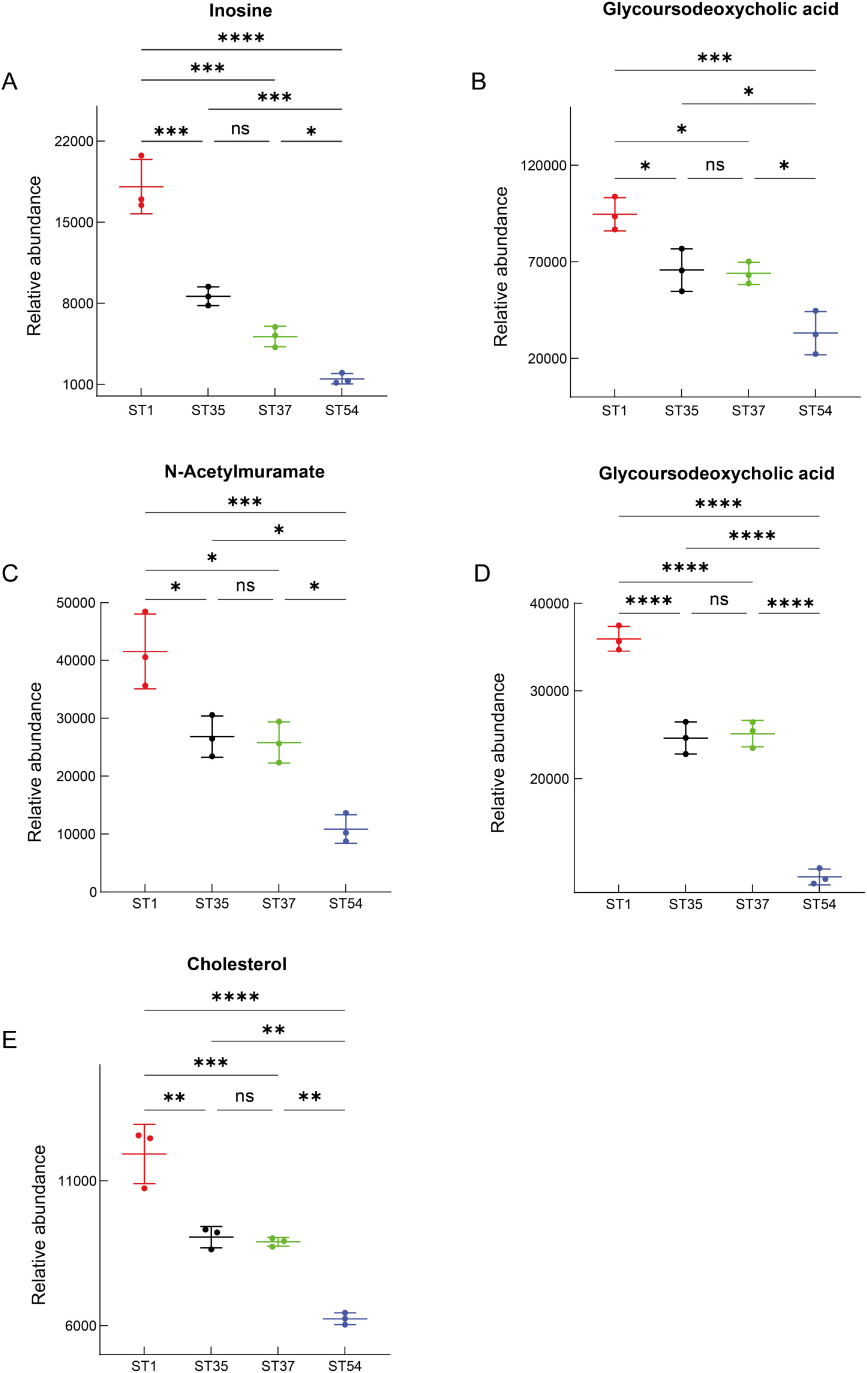
Differentially downregulated metabolites along the virulence gradient. **(A)** Inosine. **(B)** Glycoursodeoxycholic acid. **(C)** N-Acetylmuramate. **(D)** Glycoursodeoxycholic acid (additional comparison). **(E)** Cholesterol. Relative abundance levels are presented as mean ± SD. Statistical significance is indicated as ns (not significant), *P < 0.05, **P < 0.01, ***P < 0.001, ****P < 0.0001. *P*-values were adjusted for multiple testing using the Benjamini-Hochberg false discovery rate (FDR).

By integrating databases such as Metaboanalyst 6.0 and KEGG, we further identified 13 potential biomarkers with the highest biological significance associated with the virulence gradient of CDI. Among these, the following were upregulated: isomangiferin, ginsenoside ro, glycocholic acid, lactic acid, 5-aminovalerate, isovalerate, isobutyrate, and alpha-aminobutyric acid (Figure 7); and downregulated compounds included Inosine, glycoursodeoxycholic acid, n-acetylmuramate, n-acetylglucosamine, and cholesterol (Figure 8). These virulence-associated trends across strains are visualized in Figures 7, 8, in which strains are arranged from low to high virulence.

According to the MetaboAnalyst 6.0 database, the above 13 differentially expressed metabolites were primarily enriched in the following pathways: Primary bile acid biosynthesis, pyruvate metabolism, glycolysis or gluconeogenesis, cysteine and methionine metabolism, steroid biosynthesis, amino sugar and nucleotide sugar metabolism, purine metabolism, and steroid hormone biosynthesis.

## 4 Discussion

This study constructed non-targeted metabolomic profiles based on four representative *C. difficile* strains *(ST1, ST35, ST37, ST54)*, revealing clear hierarchical differences in metabolite composition across these strains along a virulence gradient. Multivariate statistical and pathway enrichment analyses revealed significant reprograming in high-virulence strains *(ST1)* compared to low-virulence strains *(ST54)* across pathways including bile acid metabolism, fermentation, membrane lipid and cell wall precursor synthesis, and nicotinic acid/nicotinamide energy metabolism. Thirteen candidate metabolites were ultimately identified as potential markers for distinguishing strain virulence. These findings not only expand the knowledge base on *C. difficile* virulence regulation mechanisms but also hold potential for translational applications as diagnostic or typing tools.

### 4.1 Rewiring of bile acid metabolism and virulence regulation

We detected a marked upregulation of glycocholic acid and downregulation of glycoursodeoxycholic acid as bacterial virulence increased across strains, suggesting bile acid composition differences among virulence strains may be closely linked to spore germination and pathogenic potential. Previous studies have demonstrated that primary bile acids such as taurocholate and cholate can significantly induce spore germination in *C. difficile*, while certain secondary bile acids or conjugated forms like chenodeoxycholate act as potent competitive inhibitors. These inhibitors suppress taurocholate-induced germination and reduce virulence expression in strains (Sorg and Sonenshein, 2010). Additional studies demonstrate that different strains exhibit varying sensitivities to secondary bile acids, with these differences correlating to strain virulence and intestinal colonization capacity (Thanissery et al., 2017). Therefore, we hypothesize that in highly virulent strains, the biosynthesis or tolerance mechanisms for primary bile acids are enhanced, while the production or action of secondary or conjugated bile acids is weakened. This bile acid remodeling may confer an advantage to these strains during spore germination and early proliferation stages within the host. Future experiments could validate the functional role of the bile acid profile by measuring the expression levels of key bile acid conversion enzyme genes in strains and comparing spore germination rates and toxin production in the presence of identified primary/conjugated/secondary bile acids.

### 4.2 Fermentation metabolism and energy metabolic reprograming

In this study, as strain virulence increased, fermentative metabolites including lactic acid, isovalerate, isobutyrate, and α-aminobutyric acid were consistently upregulated. This suggests these strains may accelerate or enhance amino acid fermentation and short/branched-chain fatty acid production to meet their high metabolic demands and toxin synthesis requirements. Previous literature indicates that exogenous short-chain fatty acids (SCFAs) like butyrate inhibit *C. difficile* growth in the environment while promoting spore formation and toxin release, demonstrating SCFAs’ dual role in regulating strain survival and virulence (Pensinger et al., 2024); Concurrently, reviews suggest SCFA levels and alterations in the intestinal metabolic environment often correlate inversely with CDI severity. High SCFA concentrations may inhibit strain proliferation but potentially drive virulence gene expression to counter competitive pressures (Gregory et al., 2021). Based on our findings, the upregulation of such fermentation products may not only reflect metabolic resource reallocation under resource-limited or competitive conditions but also trigger virulence-related regulatory pathways. These mechanisms may include altering ATP/NADH supply, inducing quorum sensing, or activating stress defense responses.

### 4.3 Membrane lipid rearrangement and cell wall precursor metabolism

Our results suggest enhanced membrane lipid–associated metabolic activity in highly virulent strains, concurrent with downregulation of cell wall precursors like n-acetylmuramate and n-acetylglucosamine. This suggests a potential metabolic trade-off between membrane structure and cell wall synthesis. In a recent study, the synthases UgtA and UgtB for crucial glycolipids were found to dominate the membrane lipid composition of *C. difficile*. Approximately 50% of the membrane’s polar lipids are glycolipids, an exceptionally high proportion compared to many other bacteria, indicating the particular importance of membrane lipid structure for the survival and pathogenicity of *C. difficile* (Zbylicki et al., 2025). Cell wall precursor synthesis pathways play a critical role in bacterial growth, morphological maintenance, and host defense evasion (Garde et al., 2021). Previous studies have shown that when cell wall synthesis is inhibited or resources are diverted to other metabolic or virulence-related processes, bacterial cell wall integrity and secretory system function may be compromised (Peyraud et al., 2016). Based on our observed downregulation trends, we hypothesize that highly virulent strains may suppress or dilute intermediates in the cell wall synthesis pathway while reconfiguring membrane lipids to optimize secretion systems or membrane protein localization, thereby facilitating toxin secretion, adhesion, or immune evasion.

### 4.4 Niacin/nicotinamide metabolism and redox state

NAD + serves as a key coenzyme for multiple dehydrogenases and redox reactions. Its availability influences bacterial metabolic pathway selection between oxidative metabolism and fermentation, which directly impacts bioenergetics, stress responses, and secondary metabolism capacity (Neumann-Schaal et al., 2019). In *C. difficile*, redox regulatory proteins and associated metabolic pathways have been demonstrated to link NAD + regeneration, fermentative metabolism, and toxin production. Consequently, alterations in nicotinic acid/nicotinamide metabolism provide plausible molecular mechanisms for understanding how highly virulent strains regulate virulence at the metabolic level (Bouillaut et al., 2019).

### 4.5 Potential and application prospects of candidate markers

The 13 metabolites identified in this study span multiple pathways, including bile acids, short-chain and branched-chain fatty acids, membrane lipids, and cell wall precursors. Their consistent variation along the virulence gradient suggests these metabolites hold potential as markers for *C. difficile* virulence typing. Bile acid metabolism is closely linked to *C. difficile* spore germination and growth. Primary bile acids like taurocholate induce germination, whereas secondary bile acids such as deoxycholate and lithocholate exert inhibitory effects (Sorg and Sonenshein, 2010; Giel et al., 2010). Short-chain fatty acids (SCFAs) like butyrate and isovalerate not only influence energy metabolism but also regulate toxin expression and spore formation (Giel et al., 2010). Furthermore, alterations in cell membrane lipid composition and cell wall precursor metabolism can affect membrane protein localization, secretory system efficiency, and tolerance to host defenses, thereby modifying virulence levels (Brown et al., 2021). These findings suggest that increased virulence may be accompanied by reprograming of energy allocation and membrane structural remodeling. Future studies could validate the diagnostic value of these metabolites through targeted LC–MS/MS quantification, animal models, and clinical samples, while utilizing ROC curves and machine learning models to assess their typification or predictive capabilities (Bea-Serrano et al., 2025).

### 4.6 Steroid biosynthesis and sulfur–amino acid metabolism

Along with the bile acid and energy metabolism, cysteine and methionine metabolism and steroid biosynthesis were also identified in the pathway enrichment analysis as the virulence-related metabolic programs (Dubois et al., 2016; Kies and Hammer, 2022). Cysteine and methionine metabolism is regarded as the key factor of redox homeostasis, methyl-group transfer and sulfur-dependent enzyme functioning, which are extremely important to the development of anaerobic bacteria and the mechanisms of withstanding stress (Tikhomirova et al., 2024). Heightened flux via sulfur amino acid metabolism could help in the production of glutathione, S-adenosylmethionine, and additional cofactors, which contribute to a higher tolerance of oxidative stress and the regulation of virulence gene expression (Dubois et al., 2016).

Steroid biosynthesis, which is a host-associated route, is tightly coupled to bacterial alteration and use of derivatized substrates of sterols and bile acids in the gut (Winston and Theriot, 2020). Host enzymes synthesize cholesterol-derived steroids in *C. difficile* into bile acids which are then altered by bacterial metabolic processes (Vinay et al., 2025). Thus, enhanced biosynthesis of steroids-associated metabolites in the highly virulent strains is consistent with the remodeling of bile acid pools, and it may also be a contributing factor to the increased availability of primary bile acid derivatives, which fosters spore germination and growth (McMillan and Theriot, 2024). This metabolic connection presents a mechanistic connection between steroid linked metabolites and the virulence correlated bile acid markers of this study.

### 4.7 Clinical and biological background of the four *C. difficile* lineages

*RT027/ST1* belongs to clade 2 and is widely recognized as a hypervirulent lineage (Orozco-Aguilar et al., 2020). It has been repeatedly associated with increased toxin A and B production, the presence of the binary toxin CDT, enhanced sporulation, and higher rates of recurrence, complications, and mortality in patients with *CDI*. Both clinical cohort studies and experimental infection models have demonstrated that *ST1* strains exhibit superior colonization, persistence, and pathogenicity compared with most other lineages (Collins et al., 2018).

In contrast, *RT012/ST54* is generally considered a low-virulence lineage. Clinical isolates of ST54 are associated with milder disease, lower toxin activity, and reduced inflammatory responses in host tissues, and are frequently recovered from patients with less severe CDI (Di Bella et al., 2024).

*RT046/ST35* and *RT017/ST37* represent intermediate-virulence lineages. These strains typically show moderate toxin production, sporulation capacity, and disease severity in clinical cohorts, falling between the extremes represented by *ST1* and *ST54* (Luo et al., 2024). In epidemiological studies from Shandong Province, China, where the strains used in this work were originally isolated, *ST35* and *ST37* were associated with intermediate clinical severity scores, whereas *ST1* showed the highest severity and *ST54* the lowest.

Therefore, the four strains analyzed in the present study represent a biologically and clinically validated virulence gradient *(ST1* > *ST35* ≈ *ST37* > *ST54)*, rather than an arbitrary ranking (Byun et al., 2019). The metabolomic differences identified here can thus be interpreted as molecular correlates of these well-established strain-specific virulence phenotypes.

### 4.8 Limitations and future directions

The great aim of this paper was to describe intrinsic, strain-specific metabolic diversity in *C. difficile* isolates with different virulence backgrounds of the bacteria in controlled and standardized conditions. A deliberate choice of an *in vitro* metabolomics platform was thus made so as to reduce host effects and allow a closer comparison of metabolic phenotypesin bacteria.

Although *in vitro* culture conditions allow control over strains and growth environments, this also implies the absence of host factors (immune responses, intestinal barriers, diverse microbial interactions) and the complexity of the actual bile acid pool and nutrient competition environment in the gut. Besides that, host and microbiota-produced bile salt hydrolases (BSHs) and other bile acid-modifying enzymes have the potential to shape bile acid profiles significantly *in vivo* further impacting *C. difficile* spore germination and growth. These factors may lead to differences between metabolic profiles observed *in vitro* and *in vivo*. In particular, host metabolism, immune responses, diet, and microbiota composition can substantially influence stool metabolomic profiles, which were beyond the scope of the present strain-focused analysis. Future studies integrating paired stool metabolomics and correlation analyses with bacterial metabolic signatures will be necessary to establish host-pathogen metabolic linkages.

Furthermore, while non-targeted LC-MS methods cover a vast number of metabolites, their identification relies on database matching and spectral libraries, leaving open the possibility of isomer confusion or annotation errors. Consequently, structural and quantitative confirmation of some candidate metabolites requires reference standards and isotope labeling. Moreover, while this study identified associations between candidate metabolites and virulence gradients, it did not directly measure toxin release, biofilm formation, or pathogenicity differences in host infection models. Thus, causal roles for these metabolites cannot be definitively asserted. Future studies should incorporate functional experiments (metabolite supplementation or depletion, knockout/overexpression of key metabolic enzymes) to assess the impact of these metabolites on virulence-related phenotypes, as well as track their dynamic changes in animal models or human clinical samples.

## 5 Conclusion

This study employed non-targeted metabolomics to conduct a systematic analysis of metabolic differences among *C. difficile* strains exhibiting varying virulence profiles. It revealed significant metabolic reprograming in highly virulent strains, particularly in amino acid fermentation (especially branched-chain amino acids), organic acid metabolism, and lipid metabolism. Combined with virulence and pathway enrichment analyses, the results suggest that metabolic status may represent a key dimension influencing bacterial virulence. Specifically, elevated abundances of branched-chain amino acids and their downstream fermentation products in highly virulent strains indicate that these strains may enhance pathogenicity by prioritizing energy acquisition from these substrates or disrupting the microbial community. Concurrently, reduced levels of several intermediate metabolites in lipid metabolism within low-virulence strains may reflect their compromised capabilities in membrane construction, signal transduction, or stress responses. Collectively, this study provides the first metabolomic perspective on the potential link between metabolism and bacterial virulence across strains of differing virulence, establishing a theoretical foundation for developing metabolism-targeted strategies against *C. difficile*. Future studies should validate these metabolic markers in *in vivo* models and investigate their potential as biomarkers for predicting strain virulence or guiding personalized treatment. Furthermore, given that metabolic pathways can be controlled through interventions such as substrate antagonism, metabolic modulators, and microbiome restoration, this metabolomics findings may offer novel approaches for suppressing CDI and recurrence.

## Data Availability

The raw data supporting the conclusions of this article will be made available by the authors, without undue reservation.

## Footnotes

1
http://pubmlst.org/cdifficile/

2
https://webribo.ages.at/
