# Supplementary material for: Non-targeted metabolomics reveals metabolic signatures associated with Clostridioides difficile virulence
Source: Front Microbiol. 2026 Feb 26;17:1731048. doi: 10.3389/fmicb.2026.1731048 (PMC12979433; doi:10.3389/fmicb.2026.1731048)
Supplement: Supplementary file 1 [file Table_1.docx]

**Supplementary Table 1** Clinical severity-based classification and molecular typing of C. difficile strains used in this study

| **MLST type** | **Ribotype** | **Strain source** | **Clinical severity category** |
| --- | --- | --- | --- |
| ST1 | RT027 | Laboratory storage | High17 |
| ST35 | RT046 |  | Moderate |
| ST37 | RT017 |  | Moderate |
| ST54 | RT012 |  | Low |

*Note.* All strains listed in this table were originally isolated from patients with clinically confirmed C. difficile infection at Zibo Central Hospital (ZCH) and the Affiliated Hospital of Qingdao University (AHQU) and subsequently preserved in laboratory storage. The disease severity classification shown here is based on a previously established clinical cohort in which comprehensive severity scoring was performed using patient symptoms, laboratory indicators, and clinical outcomes, following internationally accepted criteria. These data were reported in a published molecular epidemiology study of the same strain collection (DOI: 10.2147/IDR.S152724). Based on those clinically derived severity scores, the strains were grouped into a virulence gradient for the present study: *ST1* *(RT027)* as the highest-severity lineage^1^, *ST35 (RT046)* and *ST37 (RT017)* as intermediate-severity lineages, and *ST54 (RT012)* as the lowest-severity lineage. This clinically informed classification was used throughout the metabolomic analyses to define trends “with increasing virulence”.

1. Orozco-Aguilar, J. *et al.* In vivo animal models confirm an increased virulence potential and pathogenicity of the NAP1/RT027/ST01 genotype within the Clostridium difficile MLST Clade 2. *Gut Pathog* **12**, 45 (2020).
